# Assessment of Factors Affecting the Preference of Pain Medicine Subspecialty Choices and Training Course in Turkey: A Cross-Sectional Survey Study

**DOI:** 10.4274/TJAR.2023.221071

**Published:** 2023-08-18

**Authors:** Tural Bayramov, Halil Çetingök, Gül Köknel Talu

**Affiliations:** 1 Clinic of Algology, Kent Hospital, İzmir, Turkey; 2 Department of Anaesthesiology, Reanimation and Pain Medicine, İstanbul University, İstanbul Faculty of Medicine, İstanbul, Turkey

**Keywords:** Medical fellowship, medical subspecialty training, pain doctor, pain management, pain medicine

## Abstract

**Objective:**

The aim of this study is to assess the factors affecting the preference for the Pain Medicine subspecialty and the fellowship training programs by the pain specialists who have completed or continued the Pain Medicine fellowship training program from 2014 to 2021 in Turkey.

**Methods:**

The study was conducted in October 2020 and March 2021. By reaching out to the pain specialists who completed their fellowship or had been continuing their training by getting the right to receive a Pain Medicine fellowship. Via e-mail or WhatsApp application, an e-questionnaire link was sent to the participants, and data were collected on demographics, factors affecting the choice of Pain Medicine subspecialty, level of realization of the expectations during the training course and the level of proficiency in the field of pain specialization. Data analysis was performed using IBM SPSS Statistics 20.0 software, and tests were considered statistically significant if *P* < 0.05.

**Results:**

Participants reported that the factors that most affected their preferences were personal interest (55.1%), more comfortable working conditions (43.6%), and interest in an academic career (38.5%). Seventy-six participants answered the level of realization of expectations about performing interventional pain procedures using ultrasound imaging, and 31.6% reported that their expectations were not met, and 25% reported that their expectations were partially realised.

**Conclusion:**

We hope that our findings will lead to improving Pain Medicine subspecialty training programs, upgrading standards, and more comprehensive studies on these issues.

**Main Points**

• In 2008, Pain Medicine (Algology) was defined as a subspecialty requiring a two-year education under Anaesthesiology and Reanimation, Physical Therapy and Rehabilitation, and Neurology departments in Turkey.

• Taking subspecialty examinations annually by the Measuring, Selection and Placement Center in Turkey since 2014, specialists can be qualified as Pain Medicine (algology) doctors.

• Personal interest (55.1%), more comfortable working conditions (43.6%), and interest in an academic career (38.5%) were the factors most affecting the preference for Pain Medicine (Algology) in our study.

• The area where the participants’ expectations were met the most during the subspecialty training was the interventional procedures under fluoroscopy.

• Anaesthesiology and Reanimation departments are more integrated into Pain Medicine (Algology) fellowship program and therefore contributes more.

• Ultrasound is an essential tool for examining and treating pain patients in the practice of Pain Medicine, and this subject should be included more extensively in training programs.

## Introduction

The first clinic for treating patients suffering from chronic pain was established by anaesthesiologist E. Rovenstine at Bellvue Hospital, New York, before World War II. After 1947 the idea spread to Europe; the first pain clinic was opened at University College Hospital in London, followed by other institutions in a short time.^1^

International Association for the Study of Pain (IASP) was founded in 1974 and was the first multidisciplinary and international pain association.^1^ One year later, the first issue of the “Pain” journal was published.^2^ The establishment of other national pain societies followed the establishment of the IASP. The first Pain Unit in Turkey was established in 1986 at İstanbul University, İstanbul Faculty of Medicine, and the Turkish Algology-Pain Society was established in 1987.^3^ In Turkey’s Medical Specialization Board (MSB) published in 2001, Pain Medicine (Algology) subspecialization was only for anaesthesiologists. In the MSB of Turkey published in 2008, Pain Medicine was defined as a subspecialty requiring a two-year education in Anaesthesiology and Reanimation, Physical Therapy and Rehabilitation, and Neurology departments. The content of the subspecialty of Pain Medicine was determined by the Curriculum Commission convened in 2010 and organised by the Ministry of Health of Turkey. In 2011, the Ministry of Health evaluated past studies and experiences of doctors who applied and approved the first Pain Medicine (Algology) specialists in Turkey and were given specialization diplomas. Currently, there are 27 Pain Medicine training programs in Turkey whose protocols have been accepted since 2014.^3^

To be able to be admitted to a program qualifying for Pain Medicine fellowship in Turkey is determined according to the success of the subspecialty exam and staff preferences after completing education in Physical Therapy and Rehabilitation, Neurology, or Anaesthesiology and Reanimation residency. Usually, participants with the highest scores succeed in becoming Pain Medicine fellows. The duration of the training is two years, and the main hallmarks are determined in accordance with MSB in the form of rotations in the departments of Anaesthesiology and Reanimation, Physical Therapy and Rehabilitation, Neurology and Psychiatry during the training process of the fellows.

In the last decade, interest in the subspecialty of Pain Medicine and the number of doctors receiving specialization training in this field has increased in Turkey. Despite this, there is no published data on the preferences, training and post-training career period of pain specialists in this field.

The aim of this study is to assess the factors affecting the preference of pain management subspecialty and training courses by the pain specialists who have completed or continue the Pain Medicine fellowship program from 2014 to the present. The secondary gain of the study is to determine the programs’ low conditions and lacking facilities in the educational period, which would help improve Pain Medicine fellowship programs and training processes based on the results of the study.

## Methods

A 19-item e-questionnaire consisting of 6 sections (Supplementary File 1) was applied to physicians who were continuing or have completed their fellowship program in Pain Medicine (Algology) using Google Forms (Mountain View, California, USA).

The ethical approval of the İstanbul University, İstanbul Faculty of Medicine Clinical Research Ethics Committee was obtained for the study (no: 2020/1171, date: 25/09/2020). This study has been reported according to the principles in the Strengthening the Reporting of Observational Studies in Epidemiology (STROBE) guide.

The study was carried out in the period covering October 2020 and March 2021. It was carried out by reaching out via e-mail or WhatsApp application to the pain specialists who completed their fellowship or who had been continuing their training by getting the right to receive Pain Medicine fellowship training as a result of the annually taken subspecialty exam in the period from 2014 to the present in Turkey.

An e-questionnaire link was sent to the participants, who were asked to fill it out. Contact information of pain specialists and fellows was obtained through the Turkish Algology Association.

The e-questionnaire link was sent to 114 physicians four times, and 78 participants filled out the e-questionnaire. The first two parts of the e-questionnaire contained information about the participant’s e-mail address, the date of filling out the questionnaire and the consent form; the 3^rd^ part contained demographic information about the participant; the 4^th^ part contained the factors affecting the choice of Pain Medicine subspecialty. The 5^th^ part had the level of realization of the expectations during the training course, and the 6^th^ part was the level of proficiency of the participants who have completed the training course. Likert scale was used for evaluation in the 4^th^ and 5^th^ sections of the questionnaire. And blank spaces were left in sections 4, 5 and 6 for additional assessments of the participants (see Supplementary File 1).

The questionnaire was created after interviewing a Pain Medicine fellowship program director, based on the surveys conducted among Anaesthesiology and Reanimation residents in Turkey and Canada and Physical Therapy and Rehabilitation residents in Turkey.^4,5,6^ Eight Pain Medicine fellows participated in the piloting to ensure that the questionnaire was understandable and complete.

### Statistical Analysis

Shapiro-Wilk test was used to determine whether the distributions of continuous variables were normal or not, and the Levene test was used to evaluate the homogeneity of variances and mean, percentage distribution and standard deviation values were calculated using descriptive statistical methods. Independent Samples t-test or ANOVA test was used to compare parametric data. Cronbach’s alpha value was used to measure the internal consistency of a set of survey items. Data analysis was performed using IBM SPSS Statistics 20.0 software (IBM Corporation, Armonk, NY, USA), and tests were considered statistically significant if *P* < 0.05.

## Results

The number of participants in our study was 78 pain physicians. Response rate was 68.4%. Thirty-eight (48.7%) of the participants were female, and 40 (51.3%) were male. The mean age of the participants was 37 years (SD=4), the youngest 31 y, and the oldest 47 y.

The training profile of the participants is shown in Table 1.

Pain Medicine was the first choice of all participants, except for 5 (6.4%) participants, at the stage of subspecialty preferences after the subspecialty exam. It was seen that 4 participants whose first choice wasn’t Pain Medicine were Neurology, and 1 participant was Physical Therapy and Rehabilitation specialists.

A 4-point Likert scale was used to determine the factors affecting the Pain Medicine subspecialty preferences: 0 - no effect, 1 - little effect, 2 - moderate effect, and 3 - high effect. This section was composed of 11 items, and if any other condition affected the participant’s choice, it was reported that it should be indicated together with the impact score (Table 2).

The Cronbach α value was calculated as 0.693 for this evaluation, which included 11 items. The factors affecting the preference for the algology subspecialty (*P*=0.21) and the groups with different main specialities (*P*=0.34) were compared, and no statistically significant difference was found in either comparison.

Participants reported the three factors that had the most influence on their preferences, in order: personal interest (55.1%), more comfortable working conditions (43.6%), and interest in an academic career (38.5%). It was observed that the most negligible affecting factor was the influence of colleagues.

A 4-point Likert scale was used to determine at what level the different expectations of the participants were realised in the Pain Medicine fellowship training course: 0 - my expectations were not realised, 1 - my expectations were partially realised, 2 - my expectations were fully realised, 3 - above my expectations. This section covers nine areas, and if any other condition affects the participant’s choice, it was reported that they should specify it with the impact score.

The Cronbach α value was calculated as 0.730 for this evaluation, which included nine items. The results of the level of realization of expectations in the subspecialty training course are shown in Table 3.

The level of realization of expectations in the Pain Medicine subspecialty training process was compared between male and female participants (*P*=0.17) and the groups with different main specialities (*P*=0.77), and no statistically significant difference was found in both comparisons.

The level of realization of expectations in the training course, was compared the participants from university hospitals and training-research hospitals, and no statistically significant difference was found (*P*=0.28).

The three areas in which the participants’ expectations were met the most during the training course were: practice on fluoroscopy-guided interventions, Anaesthesiology and Reanimation rotation, and outpatient practice. The area where the least expectations were realised was reported as the psychiatry rotation (Table 3).

**Figure 1:**
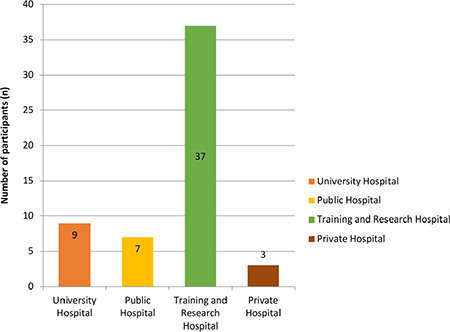
Hospitals where pain specialists work after subspecialty training.

Among the participants, 56 people completed the algology subspecialty training and the distribution of hospitals they work are shown in Figure 1.

**Figure 2:**
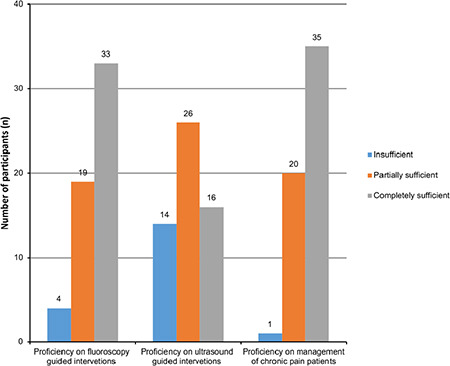
Proficiency in Pain Medicine right after completing subspecialty training.

Just after completing the subspecialty training programme, the participants were asked to evaluate themselves in X-ray and ultrasound (US)-guided interventional pain procedures and the management of the pain patient. Although the participants mostly found themselves thoroughly competent (58.9%) in fluoroscopy-guided interventional procedures and patient management with pain, 46.4% of the participants found themselves partially competent in US-guided interventional procedures, while 25% reported that they were incompetent (Figure 2).

**Figure 3:**
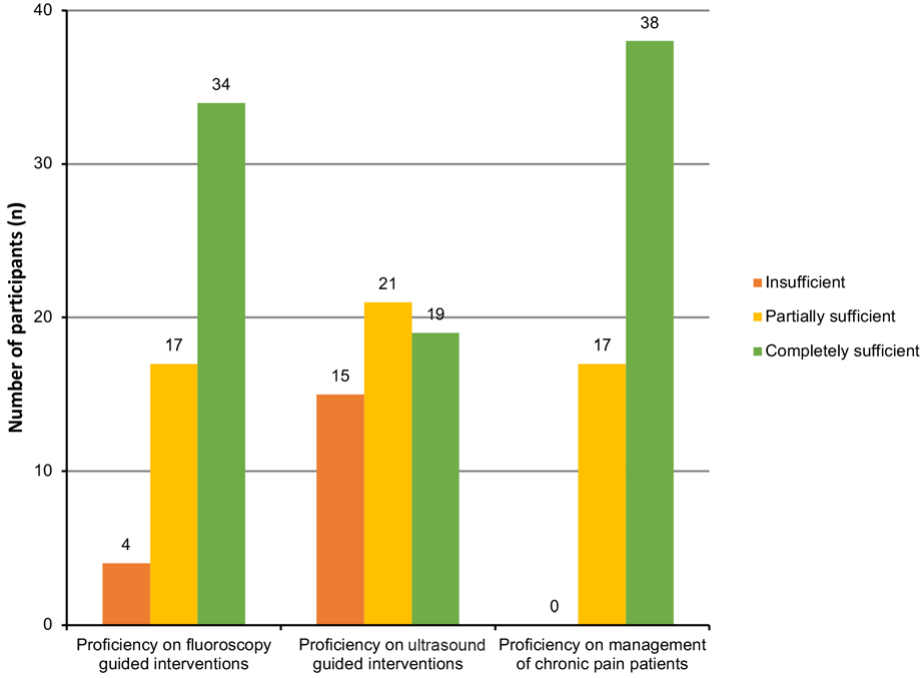
Proficiency in Pain Medicine after completing the subspecialty training and working for at least 6 months.

In the questionnaire, the participants were asked again what level of proficiency they gained after working as a pain specialist for at least six months on the same subjects. One participant did not evaluate because he worked for less than six months; the other 55 participants made their evaluations. The results of the evaluation are shown in Figure 3.

After working for at least six months, the participants improved themselves on US-guided interventional procedures and the rate of those who found them thoroughly competent increased from 28.6% to 34.5%.

## Discussion

In our study, the factors affecting the preference for Pain Medicine subspecialty in Turkey by physicians who have completed or are continuing their training in pain medicine, the expectations during the fellowship and the level of self-proficiency of the physicians in the post-fellowship period were evaluated. As far as we know, there has been no previous study in Turkey investigating which factors affect the career choices of Pain Medicine physicians, their expectations, and the Pain Medicine fellowship training process and beyond with 68.4% response rate. We found that the most important factor in the preference of a Pain Medicine specialty is personal interest, and learning the procedures performed with fluoroscopy during the training process meets the expectations the most. And during the training of fellows, Anesthesiology and Reanimation departments are more integrated into the Pain Medicine (Algology) fellowship program and therefore contribute more.

In a study conducted in Turkey by Izgi et al.^4^ among 284 Anaesthesiology and Reanimation residents, it was reported that 35.2% of the participants planned a subspecialty training after their residency, and 2/3 of this group planned Pain Medicine. In addition, among those who decided to take a subspecialty training, the most common factors affecting this decision were working in a better place during compulsory service (47.2%), improving earning potential (43.1%) and personal interest (40.4%). Sena et al.^6^ in a study with 181 physical therapy and rehabilitation residents in Turkey, only 9.4% of the participants reported that they were planning to get Pain Medicine subspecialty fellowship program. In this study, the most common factors affecting this decision were prestige (social status) (54.7%), interest in an academic career (50%) and the possibility of doing compulsory service in a better place (34.4%) among residents who were planning to take a subspecialty training.^6^ In our study, participants reported that the factors that most affected their preferences were personal interest (55.1%), more comfortable working conditions (43.6%) and interest in an academic career (38.5%). Only 29.9% of the participants reported that the score obtained from the subspecialty exam was one of the most influential factors. These results show that the participants think that the working conditions in their primary speciality in Turkey are more difficult than in Pain Medicine and that academic career opportunities are more in the field of Pain Medicine. This situation is supported by the number of pain specialists (82.1%) who have completed their fellowship program and are currently working in training and research hospitals (37 specialists) and university hospitals (9 specialists). Participants were allowed to write their own opinions in the questionnaire. Pain specialists have a compulsory duty for two years after the completed fellowship, do not always have the opportunity to do their subspecialty in compulsory duty, or cannot apply all they have learned because of the lack of infrastructure and equipment. And the contributions, such as the possibility of private practice and high job satisfaction among the influential factors of some participants drew attention.

In the section about the realization of expectations during the fellowship, 72 participants reported that they completed the Psychiatry rotation, and 31.9% of the participants reported that their expectations were not fulfilled. Only 4 (5.6%) participants gained an experience beyond their expectations during the psychiatry rotation. These results suggest a need to integrate psychiatry departments more into the process and standardize the training programs in rotations in hospitals that provide Pain Medicine fellowship programs. The rotation with the highest fulfilment of expectations was the Anaesthesiology and Reanimation rotation. This result shows us that Anaesthesiology and Reanimation departments are more integrated into the Pain Medicine fellowship program and therefore contribute more.

Nowadays, fluoroscopy remains the most commonly used technology for interventional procedures by pain specialists. The advantage of fluoroscopy is the clear visualization of the bony structures and allows precise identification of the anatomical structures necessary to perform these procedures. In our study, the area where the expectations of the participants were met the most during the subspecialty training was the interventional procedures under fluoroscopy. In pain management practice, pain specialists have started to use US imaging for examination and interventional pain procedures to reduce the use of fluoroscopy. In our study, 76 participants answered the level of realization of expectations about performing interventional pain procedures using US imaging, and 31.6% (24 participants) reported that their expectations were not met, and 25% (19 participants) reported that their expectations were partially realised. Some participants wrote that factors such as the COVID-19 pandemic, the economic difficulties of the hospital, the insufficient number of faculty members in the departments and the non-compliance with the training curriculum have an important place in the failure of the expectations.

71.8% (56) of the participants completed their Pain Medicine fellowship program. Of these participants, 4 (7.1%) reported that they found themselves insufficient in fluoroscopy-guided interventions after completing a Pain Medicine fellowship and working for at least six months. Although one participant felt inadequate at the end of the training on proficiency in managing patients with chronic pain, he could eliminate this insufficiency after working for six months. In light of these data, we see that fluoroscopy-guided interventional pain management training and management of pain patients in outpatient and inpatient are standardized throughout Turkey. At the end of the fellowship program on US in Pain Medicine practice, 14 (25%) participants reported feeling insufficient, and 26 (46.4%) partially sufficient. US not only reduces the risk of radiation exposure but is also less costly and easily transported and can be used in multiple locations. Some interventional procedures that cannot be performed with fluoroscopy can be easily performed under US guidance. Considering these advantages, it can be predicted that US will be an essential tool for examining and treating pain patients in the practice of Pain Medicine in the future, and this subject can be included more extensively in training programs.

In a survey study conducted in the USA by Asaad et al.^7^ reported that the main barriers to the use of US in the pain subspecialty training program were the lack of education of the instructors and lack of access to equipment. Although we did not get opinions from the participants on this issue in our study, we think that the same problems exist in Turkey.

The short history of Pain Medicine subspecialty training in Turkey, such as six years, and the low number of specialists (114 physicians) receiving this training are the factors limiting our study. In the Turkish and English language literature, we could not find studies conducted in other countries on the Pain Medicine subspecialty training, and this prevented us from providing the opportunity to compare the experiences of other countries. In addition, the high rate of participation in our study (68.4%), the fact that 71.8% of the participants have completed their education, and therefore they can make a more comprehensive assessment, are the advantages of our study, as it is the first research on Pain Medicine subspecialty training in the national and international arena.

## Conclusion

In conclusion, we found that the most important factors affecting Pain Medicine subspecialty preferences in Turkey are personal interests for our physicians, more comfortable working conditions and interest in an academic career. Anaesthesiology and reanimation rotation during the training process was best organized in the participants’ opinion.

In order for Psychiatry rotation to be more efficient, it should be better integrated into the Pain Medicine fellowship training process. Considering that the use of US will become more common as time progresses, there is a need to identify and solve the deficiencies in this issue in institutions providing subspecialty training on Pain Medicine, and there is a need to establish training programs on the use of US.

We hope that our study will lead to improve Pain Medicine fellowship programs, the advancement of standards and more comprehensive studies on these issues.

**Click for Supplementary File 1 access link:**

https://cms.galenos.com.tr/SolvePark/Uploads/Files/TJAR-Supplementary.pdf

## Figures and Tables

**Table 1:**
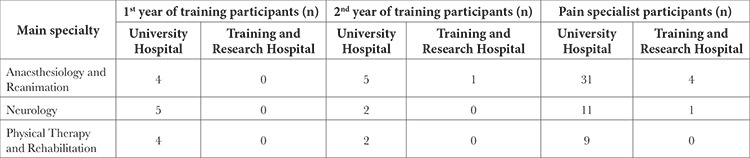
Training Profile of the Participants

**Table 2:**
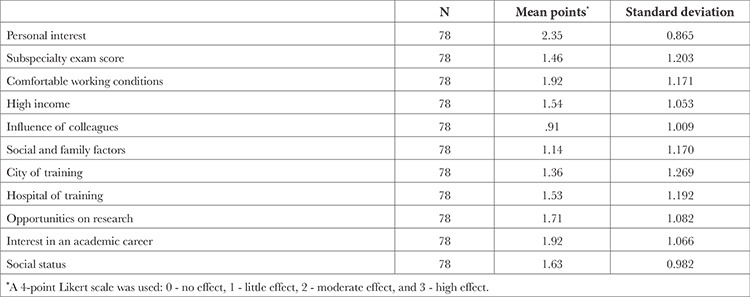
Factors Affecting the Preference of Subspecialty Pain Medicine

**Table 3:**
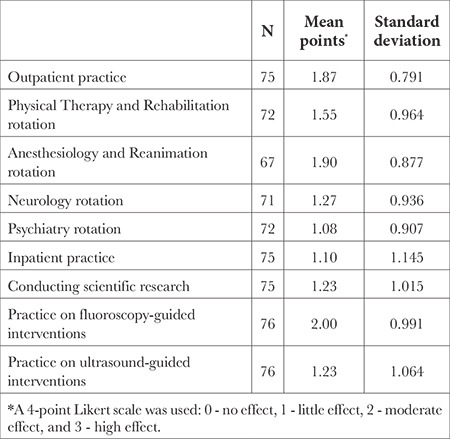
The Level of Realization of Expectations in the Subspecialty Training Course

## References

[1] Sabatowski R, Schäfer D, Kasper SM, Brunsch H, Radbruch L (2004). Pain treatment: a historical overview. Curr Pharm Des..

[2] Bonica JJ (1991). History of pain concepts and pain therapy. Mt Sinai J Med..

[3] Türk Algoloji Derneği’nin Tarihçesi. Available from:.

[4] Izgi M, Basaran B, Ankay Yilbas A, Uzun S, Pamuk AG, Kanbak M (2019). Factors affecting the preference of anesthesia residents regarding subspecialty training. BMC Med Educ..

[5] Khan J, Gilbert J, Sharma A, LeManach Y, Yee D (2015). Perspectives of anesthesia residents training in Canada on fellowship training, research, and future practice location. Can J Anaesth..

[6] Sena T, Rezvani A, Atcı AG, et al (2019). Factors influencing subspecialty training and career choices: a national survey of physical and rehabilitation medicine residents. Archives of Rheumatology..

[7] Asaad BO, Reinsel RA, DeVeaux E, Moten H, Durkin B (2014). A survey on teaching ultrasound-guided chronic pain procedures in pain medicine fellowship programs. Pain Physician..

